# Assessing Low-Intensity Relationships in Complex Networks

**DOI:** 10.1371/journal.pone.0152536

**Published:** 2016-04-20

**Authors:** Andreas Spitz, Anna Gimmler, Thorsten Stoeck, Katharina Anna Zweig, Emőke-Ágnes Horvát

**Affiliations:** 1 Northwestern Institute on Complex Systems (NICO), Northwestern University, Evanston, IL, United States of America; 2 Institute of Computer Science, Heidelberg University, Heidelberg, BW, Germany; 3 Department of Ecology, University of Kaiserslautern, Kaiserslautern, RP, Germany; 4 Department of Computer Science, University of Kaiserslautern, Kaiserslautern, RP, Germany; Universidad Rey Juan Carlos, SPAIN

## Abstract

Many large network data sets are noisy and contain links representing low-intensity relationships that are difficult to differentiate from random interactions. This is especially relevant for high-throughput data from systems biology, large-scale ecological data, but also for Web 2.0 data on human interactions. In these networks with missing and spurious links, it is possible to refine the data based on the principle of *structural similarity*, which assesses the shared neighborhood of two nodes. By using similarity measures to globally rank all possible links and choosing the top-ranked pairs, true links can be validated, missing links inferred, and spurious observations removed. While many similarity measures have been proposed to this end, there is no general consensus on which one to use. In this article, we first contribute a set of benchmarks for complex networks from three different settings (e-commerce, systems biology, and social networks) and thus enable a quantitative performance analysis of classic node similarity measures. Based on this, we then propose a new methodology for link assessment called z* that assesses the statistical significance of the number of their common neighbors by comparison with the expected value in a suitably chosen random graph model and which is a consistently top-performing algorithm for all benchmarks. In addition to a global ranking of links, we also use this method to identify the most similar neighbors of each single node in a local ranking, thereby showing the versatility of the method in two distinct scenarios and augmenting its applicability. Finally, we perform an exploratory analysis on an oceanographic plankton data set and find that the distribution of microbes follows similar biogeographic rules as those of macroorganisms, a result that rejects the global dispersal hypothesis for microbes.

## Introduction

Modern sciences like biology, economics, sociology, and even linguistics increasingly rely on the analysis of large complex networks [1–5]. Such complex networks represent entities of a given system as nodes which are linked if the corresponding entities participate in a well-defined relationship. The majority of complex networks suffer from low-intensity relationships that can be difficult to distinguish from incidental observations, i.e. false positives [6]. There are two basic ways of dealing with such a problem by applying a threshold to the measured intensity values. The first is to set a threshold which is high enough to exclude false positive links almost entirely. This approach, however, is likely to result in many false negative links, because it also removes true links with a low-intensity [7, 8]. The second approach uses a lower significance threshold which includes interactions with low intensity at the expense of adding false positive observations as well. This dilemma is similar to a common problem in learning, where sets of variables are generally not separable by a linear separator. It can thus be beneficial to increase the dimensions of the considered space, i.e. to include additional information instead of relying on a single threshold.

One possibility for such a new dimension lies in the structure of the network itself. Complex networks are highly resilient against noise in the data when links are added or removed uniformly at random [9, 10]. In this article we explore this approach and show that it is possible to tackle the problem of missing (false negative) or additional (false positive) links by evaluating similarities in the connection patterns of incident nodes. This structural similarity idea [11, 12] is based on the observation that entities that are alike tend to share parts of their immediate neighborhood. Therefore, the main assumption is that the represented relationship adheres to the notion of *homophily*, i.e. entities that are alike are more likely to be connected than entities that are not alike. While the notion of homophily has so far mainly been used in the context of social networks, it can easily be extended to networks of other entities such as proteins sharing a biological function or movies with similar genre, plot, or cast. The idea of a generalized homophily forms the basis of recommendation algorithms [13, 14], one-mode projections of bipartite networks [15, 16], and clustering algorithms [10]. It has also been used for assessing the quality of biological data, as shown by Goldberg and Roth [17]: Protein–protein interaction databases contain noisy data, since they are based on high-throughput experiments which induce many false positive observations. Furthermore, they include a large percentage of protein pairs that have not yet been tested and result in false negative interactions [18]. As an alternative to high-throughput experiments, protein–protein interactions can be observed through more reliable methods like co-immunoprecipitation. Interactions that are verified by these small-scale methods can thus be used as *ground truth*, i.e. they should be ranked high by an effective node similarity measure, independent of whether the interaction itself has ever been observed in a high-throughput experiment. Goldberg and Roth tested four different similarity measures and showed that the significance of the number of common neighbors of any two proteins with respect to a hypergeometric null model obtains the best performance, i.e. it ranked verified interactions higher than any other tested method. However, without other ground truth data sets, the analysis of Goldberg and Roth was limited to protein–protein interactions and to those similarity measures that are usually applied in biology. Here, we perform a more comprehensive study that includes a wider range of similarity measures and is based on data sets from areas as diverse as biology, sociology, and economics. The goal is to find the best similarity measure or ranking approach for assessing the quality of links that represent low-intensity relationships in a given complex network.

Our work is related to the vast body of research on link prediction (for instance [19, 20]), recommender systems (for example [14]), and the branch of network analysis that focuses on node similarity [21, 22]. Some of the proposed approaches in these areas rely on supervised learning, meaning that they require the existence of ground truth observations that include both positive and negative samples. However, in the majority of cases that are of practical relevance, such observations are not available or can only be acquired through costly experiments and surveys. Studies that face the issue of a lacking ground truth instead employ a form of unsupervised learning. Typical approaches are based on an exploratory analysis that uses scoring functions aimed at ranking positive samples higher than negative samples [23]–an approach which imposes difficulties in the evaluation of the proposed methods’ performance. In this article, we therefore use only unsupervised approaches, but evaluate their quality based on carefully constructed ground truths. This enables us to compare some of the most commonly used similarity measures and to suggest a novel methodology that outperforms them in assessing the veracity of links.

## Methods

In this section, we introduce the data sets that we use as benchmarks for evaluation and formally define the compared similarity measures before we present a new way of assessing the similarity of two nodes’ neighborhoods based on a statistical significance test of the number of their common neighbors.

### The data

We compiled data sets describing relationships in diverse complex systems and created up-to-date benchmarks (see S1 Text). The first such benchmark is for a protein–protein interaction (PPI) network based on the Database for Interacting Proteins (DIP) [24]. The ground truth is given by the manually checked, so-called *core* interactions. Based on data provided by Yang and Leskovec [25], we created a benchmark for assessing the quality of friendship networks on LiveJournal. Finally, we use product ratings by users from MovieLens and Netflix to assess the similarity of movies based on two ground truth sets, one containing movie series and the other consisting of TV series. The latter two data sets are bipartite, i.e. they contain only links between users and products. The ground truth is given as a subset of pairs of products that are similar. Table 1 describes the size of these data sets and ground truths.

**Table 1:** Data sets and ground truths.

Data set	Type	Number of nodes	Number of links	Number of GT links	GT density
PPI	not bipartite	5,078 proteins	22,148 interactions	3,543	2.75 ⋅ 10^−4^
LiveJournal	not bipartite	11,755 individuals	80,023 friendships	126,515	1.83 ⋅ 10^−3^
MovieLens Movies	bipartite	9,153 films & 15,185 users	1,077,270 ratings	450	1.07 ⋅ 10^−5^
Netflix Movies	bipartite	16,306 films & 20,078 users	2,399,429 ratings	904	6.80 ⋅ 10^−6^
Netflix TV Series	bipartite			951	7.15 ⋅ 10^−6^

Size of the used data sets and the corresponding ground truths.

Note that the data sets have different characteristics with respect to the nature of the ground truth and the number of ground truth links that are contained in the original data (see Table 2):

1. In the bipartite data sets, no ground truth links are directly contained in the network data, because the ground truth contains pairs of movies or seasons of the same TV series, while the network is a bipartite network between users and the movies they rated with at least a 4 on a scale from 1 to 5. Based on various similarity measures, 1,416,621 pairs of movies are ranked in the MovieLens Movies data set, 450 of which are part of the ground truth. In the Netflix Movies data set, 5,078,220 pairs are ranked and 904 of those belong to the ground truth. In the Netflix TV Series data set, 3,475,430 pairs are ranked and the ground truth contains 951 links.
2. By construction, the PPI network data contains all available ground truth information, which is hidden by numerous spurious interactions. Additionally, some of the observed interactions may in fact correspond to actual interactions, but have not yet been tested in small-scale experiments (not validated interactions). Furthermore, some of the actual interactions may not have been observed yet (missing interactions). In the PPI data set, there are 3,543 ground truth links and 431,324 ranked protein–protein pairs.
3. The social network of the LiveJournal data contains 19.5% of the ground truth relationships. Of all possible pairs of users, 1,440,611 are ranked by their respective similarity, of which 24,533 belong to the ground truth.

**Table 2:** Type and coverage of the data sets and ground truths.

Network type	Data set	Relationship	Ground truth	Percentage of ground truth links contained in data
biological	PPI	high-throughput and manual observation of protein–protein interactions	manually verified interactions marked as DIP *core*	100%
social	LiveJournal	declared friendship	user-defined groups	19.5%
product-rating	MovieLens Movies Netflix Movies Netflix TV Series	rating of movies by users	movie sequels seasons of TV series	0%

The data comprises three different complex systems and five ground truth data sets. The ground truth is either partially or completely contained in the not bipartite networks (LiveJournal friendship network and PPI network), but not contained in the bipartite networks (movie-rating networks).

With this selection of data sets we cover diverse real-world systems that differ not only in terms of their nature and size, but for which the compiled ground truths also vary significantly in their coverage as shown by the percentage of ground truth links that are contained in the data (see Table 2). Moreover, the mechanisms behind the formation of links in the individual networks are very dissimilar and relate in different ways to the concept of structural similarity. Therefore, these data sets pose a broad range of challenges for similarity measures that arise from different network signatures. Based on these data sets we can thus provide a more thorough evaluation of the performance of a number of similarity measures in tackling the two prominent link assessment tasks that we present in the following. The ground truth and the available data sets can be found in a zip-file (Supporting Information).

### The link assessment problem

An already quite well-researched problem is the *link prediction* problem that asks, given a static snapshot of network at time *t*, for a prediction of which of the yet unconnected node pairs will be most likely connected in the next time unit *t* + 1 [20]. The *link assessment problem* asks, given a static snapshot of network at time *t*, for an assessment of the links in it, i.e., whether the observed ones are truly existing (true positives) and whether not observed ones are indeed missing (true negatives). The problem comes in two versions: the most straightforward link assessment task aims at finding the pairs of nodes that are globally most likely to be connected. This is a key problem in experimental biology where researchers are often required to avoid arduous and expensive studies on a huge selection of agents by reducing the potentially overwhelming set of possibilities to the most probable interactions. Besides this formulation on a global level, the problem can also be addressed at a local scale by asking for the most likely links of a given node. In e-commerce or social networks, the problem of link assessment arises whenever individuals are searching for products that are tailored to their needs or suggestions of potential companions and colleagues. These two versions of the problem can be formalized as follows.

**Fig 1:**
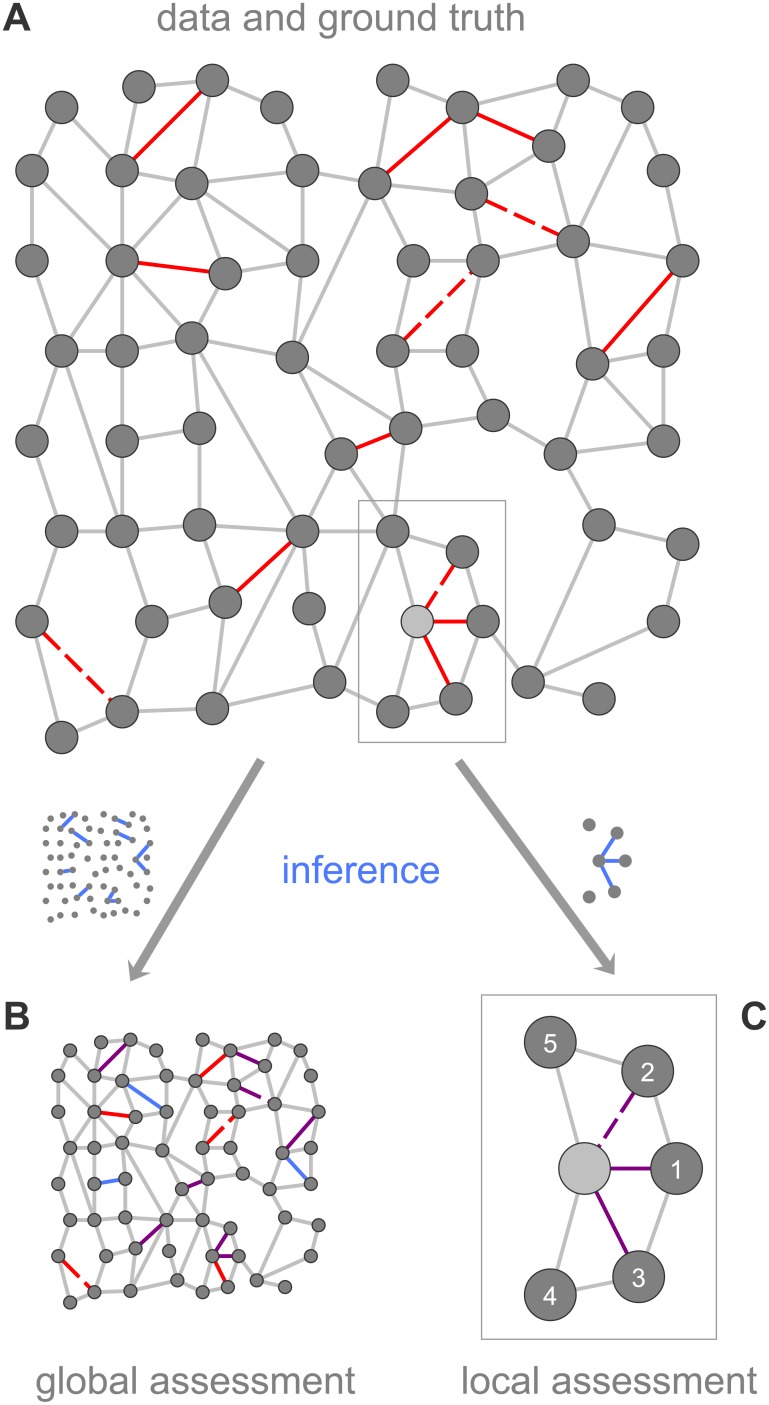
A simplified network used for the schematic illustration of the link assessment problem. (A) Given a set of ground truth links, some of them are included in the observed graph (solid red), while others are not (dashed red). Spurious observed links are colored gray. (B,C) Using a structural similarity measure, a global (local) assessment infers that the globally (locally) most similar node pairs should be connected. Inferred links are colored blue, validated links are colored violet.

Given a data set which is modelled by an undirected graph *G* = (*V*, *E*) consisting of a set of nodes *V* and a set of links *E*, as well as a ground truth data set *E*_*GT*_ ⊆ *V* × *V*, find a similarity measure s:V×V→R maps links that are contained in the ground truth to a higher value than links that are not contained in the ground truth (see Fig 1A). We can then differentiate between the global and the local link assessment problem:

1. *Global link assessment problem* (GLAP): a similarity measure *s* is said to be globally optimal if, for all (*v*, *w*) ∈ *E*_*GT*_ and all (*v*′, *w*′) ∉ *E*_*GT*_: *s*(*v*, *w*) ≥ *s*(*v*′, *w*′).
2. *Local link assessment problem* (LLAP): a similarity measure *s* is said to be locally optimal if, for all *v* in *V*, *s*(*v*, *w*) ≥ *s*(*v*, *u*) if (*v*, *w*) ∈ *E*_*GT*_ and (*v*, *u*) ∉ *E*_*GT*_.

Note that a globally optimal similarity measure is necessarily locally optimal as well, but not necessarily vice versa (see S2 Text). With a globally optimal similarity measure, all node pairs can be ranked globally based on the observed graph, and the top *k* pairs can then be included in a new graph (Fig 1B). If *k* equals the number of ground truth links *t*: = |*E*_*GT*_|, the new graph consists of only the ground truth links. In the local ranking approach, for each node *v*, the new graph contains the links which connect *v* with the nodes that are most similar to it. The number of included links *t*(*v*) equals the number of *v*’s ground truth links, i.e. *t*(*v*) := |{(*v*, *w*)∣(*v*, *w*) ∈ *E*_*GT*_}| (Fig 1C). The node similarity measures presented in the following can be used both in the GLAP and LLAP setting.

### Node similarity measures

Different scientific communities have proposed structural similarity measures for validation of experimental findings [17], recommendation systems based on data-mining [26], and computation of one-mode projections [27, 28]. In this article, we compare the performance of the most popular structural similarity measures in solving the GLAP and the LLAP (for further details see S3 Text). These measures are inherently based on the number of common neighbors *n*(*v*, *w*) of two nodes *v*, *w*:

1. The *Jaccard* index [29, 30] of a pair of nodes, which equals *n*(*v*, *w*) divided by the size of the union of their neighborhoods. The degree *d*(*v*) of a node *v* is defined as the number of its neighbors.
  $$\begin{array}{c} Jaccard\left(v,w\right):=\frac{n(v,w)}{d(v)+d(w)-n(v,w)} \end{array}$$ (1)
2. The *cosine* [31] of a pair of nodes is given by *n*(*v*, *w*) normalized by the geometric mean of their degrees:
  $$\begin{array}{c} cosine\left(v,w\right):=\frac{n(v,w)}{\sqrt{d(v)d(w)}} \end{array}$$ (2)
3. The *Pearson* correlation coefficient [32] is computed based on the adjacency vectors of the two nodes:
  $$\begin{array}{c} Pearson\left(v,w\right):=\frac{cov(v,w)}{\sigma [v]\sigma [w]}, \end{array}$$ (3)
  where *cov*(*v*, *w*) denotes the covariance of the adjacency vectors of nodes *v* and *w*, while *σ*[*v*] denotes the standard deviation of the adjacency vector of node *v*.
4. The *AdamicAdar* measure [33, 34] is defined as the weighted sum of the degrees of *v* and *w*’s common neighbors. If *N*(*v*, *w*) denotes the set of common neighbors of *v* and *w*, the measure can be defined as:
  $$\begin{array}{c} AdamicAdar\left(v,w\right):=\sum_{u\in N(v,w)}\frac{1}{log(d(u))} \end{array}$$ (4)
5. The resource allocation index (*rai*) refines the number of common neighbours by assigning highly connected neighbours less weight [35]:
  $$\begin{array}{c} rai\left(v,w\right):=\sum_{u\in N(v,w)}\frac{1}{d(u)} \end{array}$$ (5)
6. Based on a simple null model in which for each node *u*, *d*(*u*) neighbors are picked uniformly at random like in the configuration model [36], the difference between *n*(*v*, *w*) and the expected number of common neighbors [22] is also known as the *leverage* [26]:
  $$\begin{array}{c} leverage\left(v,w\right):=\frac{1}{\left|V\right|}\left(n\left(v,w\right)-\frac{d(v)d(w)}{\left|V\right|}\right), \end{array}$$ (6)
  where |*V*| denotes the number of nodes in the graph.
7. The Leicht-Holme-Newman index (*lhn*) is based on the same simple null model and is defined as the ratio between the observed and expected number of common neighbors [21], also known as the *lift* [26]:
  $$\begin{array}{c} lhn\left(v,w\right):=\frac{n(v,w)}{d(v)d(w)} \end{array}$$ (7)
8. The *p*-value of the number of common neighbors in a hypergeometric null model is called *hypergeom* [17, 37]. It is computed from the degrees of the two nodes and the total number of nodes as follows:
  $$hypergeom(v,w):=\sum_{c=n(v,w)}^{\text{min}\{d(v),d(w)\}}\frac{\left(\begin{array}{c} d(v)\\ c \end{array}\right)\left(\begin{array}{c} \left|V|-d(v\right)\\ d(w)-c \end{array}\right)}{\left(\begin{array}{c} \left|V\right|\\ d(w) \end{array}\right)}$$ (8)

Note that some of the measures have a slightly different formulation when adapted to bipartite graphs as detailed in S3 Text. In the next section, we describe the new approach to link assessment by evaluating the statistical significance of the number of common neighbors in comparison to a more realistic random graph model, the *fixed degree sequence model* (FDSM).

### A novel methodology for link assessment based on comparison with the FDSM

The leverage, the Leicht-Holme-Newman index, and the p-value of the number of common neighbors based on a hypergeometric null model compare the observed number of common neighbors with an expected value or assess the statistical significance of an observation with respect to some expected distribution. Such an expected value or the distribution of the expected values can also be obtained from a set of random graphs that have the same number of nodes and links and the same degree sequence as the observed graph [23]. Theoretical research showed that this null model, known as the *fixed degree sequence model* (FDSM), is superior to the simple random graph model deployed by the configuration model for at least some graph families [38]. It was also empirically shown, that the expected values of various graph structures in the FDSM cannot simply be approximated by other, simpler random graph models [39], such as the average clustering coefficient. However, the performance of the statistical assessment of the number of common neighbors based on their expected value in the FDSM in the link assessment task has not been investigated yet. The main contribution of this paper is the study of the quality of different test statistics deduced from a comparison of the observed number of common neighbors with those expected in the FDSM in the broader context of link assessment, both for bipartite and non-bipartite graphs.

The FDSM contains all graphs with a given degree sequence that are simple, i.e., those without multiple links and self-links. There is an algorithm with which such graphs can be built uniformly at random [40, 41]. By randomly sampling a large number of such graphs, both the expected value 〈*n*(*v*, *w*)〉 and the sample deviation *σ*[*n*(*v*, *w*)] can be approximated. Based on this, the sample *z*-score of an observed number of common neighbors *n*(*v*, *w*) is computed by the formula:

$$\begin{array}{c} z\left(v,w\right):=\frac{n(v,w)-\langle n(v,w)\rangle }{\sigma [n(v,w)]} \end{array}$$ (9)

Additionally, the same set of random graphs allows computing the empirical *p*-value, i.e., the number of sample graphs in which *v* and *w* have at least *n*(*v*, *w*) neighbors.

While in theory, the ranking of all node pairs by the *z*-score and the *p*-value should be the same, empirical research shows that this is actually not the case. This is on the one hand caused by the limited number of samples that can be drawn. In consequence, very low p-values cannot be approximated precisely. On the other hand, the statistical assessment of pairs of nodes in which both have a low degree and a comparatively high number of common neighbors is problematic because the distribution of expected values is then not well approximated by a normal curve (for further details see S3 Text). These node pairs produce a high number of 0 or very low *p*-values. As we will show below, a ranking that sorts all node pairs by their *p*-value and where ties are broken by their *z*-scores, can amend the weaknesses of both statistics and shows consistent top performances in the link assessment problem.

## Results

Having introduced the traditional node similarity measures and z* based on the proposal to use the FDSM as an appropriate null model, we now present the results of our evaluation on our benchmarks. To this end, we include both the global and local link assessment problem.

### Identifying the overall most probable links: Performance evaluation for GLAP

**Fig 2:**
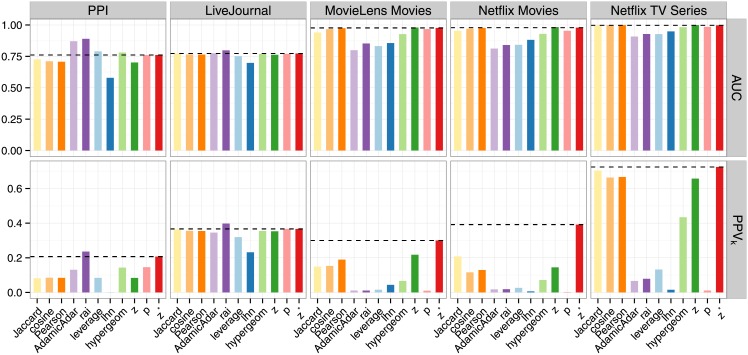
Quality of discovering the ground truth in the GLAP as quantified by the area under the ROC curve *AUC* (upper row) and the positive predictive value *PPV*_*k*_ (lower row). Based on both performance measures, z* is the only stable top-performing method for link assessment for all data sets with the exception of PPI when using the *AUC*.

For every pair of nodes with *n*(*v*, *w*)>0, we compute all of the presented similarity measures. To quantify their performance with respect to the global version of the link assessment problem, we first calculate the corresponding area under the receiver operator characteristic (ROC) curve (*AUC*). The *AUC* is a scalar performance measure that quantifies the probability that a randomly chosen ground truth link is assigned a higher score than a randomly chosen pair of nodes without a link in the ground truth. A perfect predictor achieves an *AUC* of 1, while random guessing yields a value of 0.5. As shown in Fig 2 (upper row), z* is always among the top 5 performing measures for all data sets. However, the measures that perform better than z* in some of the data sets, have an inconsistent performance overall. A ranking based on the *z*-score is the best competitor and reaches top performance in three out of five data sets, but ranks third and second to last for the other two data sets. Furthermore, the measures that outperform z* in the case of the PPI data set are the weakest predictors for the other data sets: namely *AdamicAdar*, *rai*, *leverage*, and *hypergeom*, which are well behind the other measures for the bipartite data sets MovieLens Movies, Netflix Movies, and Netflix TV Series. For all data sets besides PPI, we find that the *AUC* is not well suited to differentiate between the top-performing measures. For example, for the Netflix TV Series data set, the top 5 measures all have an *AUC* of at least 0.996. This is due to the fact that the ground truth edges are very sparse w.r.t. the number of all possible edges, i.e., the ground truth is very imbalanced (see Table 1).

The *AUC* has been shown to be an inaccurate performance measure for small and imbalanced samples [42]. In all five data sets, the imbalance is at least 2 out of 1000 (see Table 1) and for the two ground truth data sets in Netflix, we find only 7 ground truth edges per one million possible edges. Thus, we also compute a second measure, which was designed for strongly imbalanced data sets, to evaluate the quality of the predictions. Instead of aggregating over the entire range of thresholds (as in the calculation of the *AUC*), this measure quantifies the classification accuracy at a single, data set-driven threshold and evaluates the identification of the top-ranked links instead of the entire ranking. Since most applications require just a few predictions to guide experiments or make personalized suggestions, this is a sensible and practical alternative. Based on a proposal by Liben-Nowell and Kleinberg, the measure we will call hereafter *PPV*_*k*_ is defined as the *positive predictive value* among the first *k* node pairs with the highest similarities, where *k* := *t*, i.e. the number of links in the ground truth [34]. It can be shown that the *PPV*_*k*_ has a linear correlation with the sensitivity (the ability of the measure to rank the positive links high) and the specificity (the ability to rank negative links low) at this specific threshold and thus combines both performance measures into one. Fig 2 (lower row) compares the *PPV*_*k*_ values that result from the various similarity measures. The *PPV*_*k*_ differentiates between the similarity measures better than the *AUC*. Again, on the example of the Netflix TV Series data set, the top-5 measures now have values between 0.72 and 0.65, where z* identifies 72% ground truth edges among the top 951 ranked node pairs while the *z*-score ranking only identifies 0.65%. A second example also immediately reveals that the *AUC* is not well suited to quantify the performance of a link assessment method for very imbalanced data sets: while the *AUC* indicates that almost all measures perform very well in the MovieLens Movies set, the *PPV*_*k*_ shows that at most 30% of the most important top 450 predictions are ground truth edges. This is due to the fact that positive links are ranked high in comparison to most of the negative links, but are ranked low when compared to the small number of positive links, which is caused by the imbalance in the data. Thus, a measure that performs well based on the *PPV*_*k*_ and poorly according to the *AUC* is a measure for which the first *k* entries are predominantly ground truth edges, but which ranks the remaining edges from the ground truth low. Since one is usually only interested in the top predictions, the *PPV*_*k*_ is the more meaningful quality measure for imbalanced link assessment tasks. Based on the *PPV*_*k*_, z* performs best in the bipartite data sets and second-best after *rai* in the non-bipartite data sets. In the case of MovieLens Movies (A *PPV*_*k*_ of 0.30 vs 0.22), and Netflix Movies (0.39 vs 0.21) z* is even overtaking the second-best measure by a large margin; the difference of the values to *rai* in the non-bipartite data sets is 0.03 for both, the PPI data set (0.24 vs 0.21) and the Live Journal data set (0.40 vs 0.37). Note also that the performance of *rai* is below 0.08 for all bipartite data sets.

**Fig 3:**
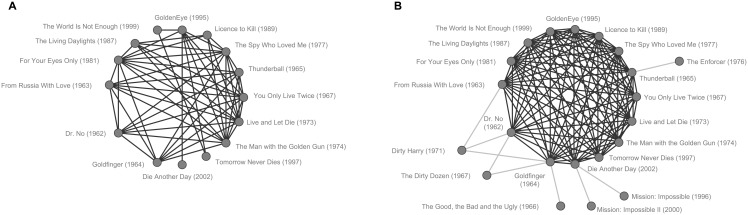
Similarities inferred by z* for the James Bond movies in the Netflix Movies ground truth. We show links between the globally most similar pairs of movies involving at least one of the Bond movies (global assessment, A) and the 14 most similar movies to each movie in the Bond series according to a local assessment (B). Duplicate edges are disregarded.

To illustrate the accuracy of the results provided by z*, in Fig 3A we show the example of the 15 James Bond movies that can be found in the Netflix data set—it is a challenging example because in the data set, the years of production of these films range from 1962 to 2002. The list of the *t* = 904 globally most similar node pairs based on z* in the Netflix Movies ground truth contains 55 of the 105 possible edges between these movies.

### Detecting links on a node-by-node basis: Performance evaluation for the LLAP

**Fig 4:**
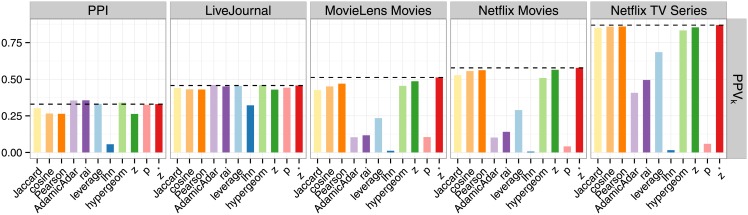
Performance of the different similarity measures in the LLAP as quantified by the *PPV*_*k*_.

Next, we evaluate the similarity measures with respect to the LLAP. Since we are interested in a certain number of the topmost predictions in this case, we again use the *PPV*_*k*_ to quantify the accuracy of the measures among the *t*(*v*) locally highest ranked links for every node *v*. Recall that *t*(*v*) denotes the number of ground truth links of node *v*. As shown in Fig 4, the measures have varying performance in solving this version of the link assessment problem as well, depending on the data set. However, z* remains the top-performer in three out of five cases, and in the LiveJournal data set the difference between its performance and the top-performer is only 0.004. In the PPIdata set, it has a performance of *PPV*_*k*_ = 0.33 with a difference of 0.03 to the top-performing measures *rai* and *AdamicAdar* (both with a *PPV*_*k*_ = 0.36).

Fig 3B depicts the result of the local assessment according to z*, illustrated again on the example of the James Bond movies from the Netflix Movies data set. The novel method finds 201 out of 210 (96%) ground truth edges. Note that, based on the quality assessment provided by the *PPV*_*k*_, all measures are significantly better in solving the LLAP than the GLAP. Accordingly, for each measure and data set, the *PPV*_*k*_ that is achieved when choosing the *t* globally highest ranked node pairs is much lower than if the *t*(*v*) locally highest ranked links of every node *v* are chosen. The reason for this is that the *PPV*_*k*_ as quality measure considers more information about the problem in the LLAP, as we use the number of ground truth edges per node for the calculations, thus making this task easier, independently of the employed similarity measure.

### The only stable top-performing method: z*

**Fig 5:**
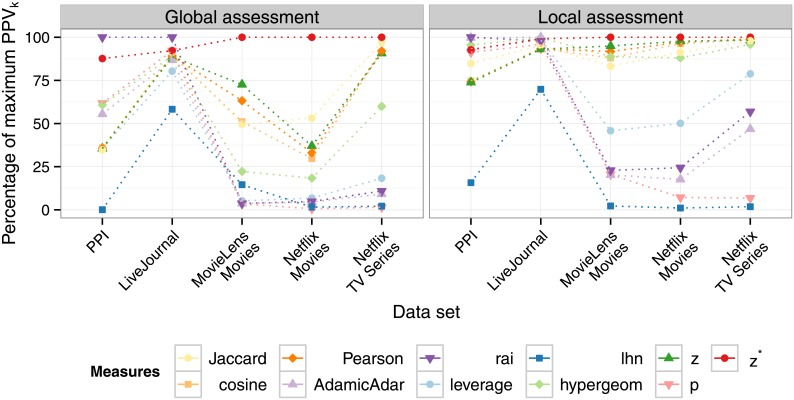
Overview of the performance of the individual measures for the considered data sets. For each measure, we show the achieved performance on the given data set as the percentage of the *PPV*_*k*_ of the top-performing measure. In both link assessment problems, z* is the most stable, top-performing similarity measure.

Regardless of the considered version of the link assessment task, none of the traditional similarity measures is consistently stable over all data sets. Fig 5 shows the *PPV*_*k*_ for each similarity measure as the percentage of the maximally achieved *PPV*_*k*_ by any of the measures on the same data set. In 6 out of 10 cases, z* obtains the maximum *PPV*_*k*_. While, e.g., *AdamicAdar* and *rai* are (near) optimal in some data sets for solving either the GLAP or the LLAP, they perform poorly in others. For example, *AdamicAdar* achieves the maximal *PPV*_*k*_ of all similarity measures on the PPI and the LiveJournal data in the LLAP, but is below 5% of the maximally achieved *PPV*_*k*_ on the MovieLens Movies and Netflix Movies data in the GLAP. Accordingly, assessing the statistical significance of the number of common neighbors with respect to the FDSM as in the newly introduced z* is the only stable, top-performing method in both the GLAP and the LLAP. The efficient combination of the *p*-value and *z*-score proposed by z* largely improves the performance of its constituent elements. For instance, when applied to the Netflix Movies data set to solve the global assessment problem, the *p*-value barely achieves 0.5% of the performance of z*, while the *z*-score obtains 37%. For a data set without further information, z* is thus the most promising method for solving the link assessment problem.

### Spatial scaling of oceanic plankton

In a final, exploratory step, we apply the top-performing measure z* to a novel oceanographic data set with no known ground truth to demonstrate how the method can be used to obtain practical insight. Thereby, we address a fundamental and controversially debated question in microbial ecology, namely biogeographic distribution patterns of unicellular microbes. The spatial (and temporal) distribution of biodiversity is an essential cornerstone in the understanding of mechanisms that generate and maintain diversity and contribute to ecosystem functioning [43]. While it can be argued that microbes are not restricted by geographic barriers (e.g. Finlay [44]), there exists evidence for spatial patterns of microbes on different distance scales (e.g. as reviewed in Martiny et al. [45]). Thus far, however, the question of spatial scaling of microbes has been difficult to address adequately and with rigor due to incomplete sample sizes and low coverage of sampling regions that were processed and analyzed in the same context. The TARA Oceans project with its global sampling [46] in concerto with high-throughput sequencing strategies produced massive data sets (up to sample saturation [47]), thus alleviating these difficulties and eliminating biases of previous analyses, which may have led to the contrasting views on spatial microbial diversity patterns (see S4 Text).

**Fig 6:**
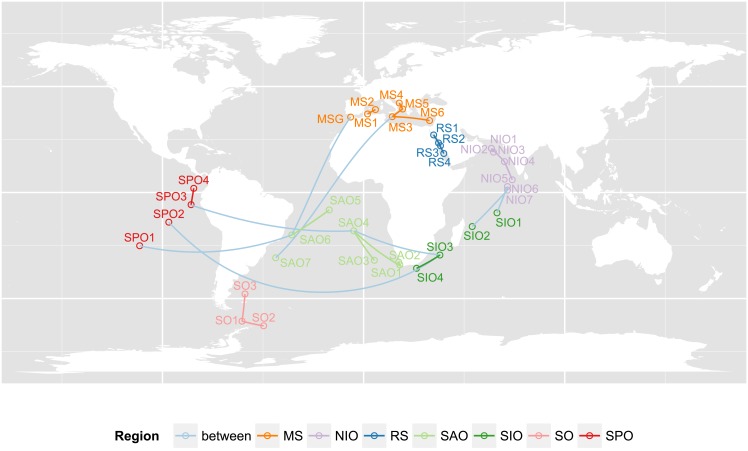
Each sample location’s ciliated plankton community (CPC) is connected to the most similar other CPC according to z* in a local ranking approach. The results suggest that the global dispersal hypothesis for microbes as postulated by Finlay [44] should be rejected. Instead, the new method shows clear evidence that microbes follow similar biogeographic rules as higher animals and plants as evidenced by Martiny et al. [45]. Shorthand notation for the oceanic regions: MS (Mediteranean Sea); NIO (North Indian Ocean); RS (Red Sea); SAO (South Atlantic Ocean); SPO (South Pacific Ocean); SIO (South Indian Ocean); SO (Southern Ocean). The direction of edges is disregarded.

We applied z* to *ciliated plankton communities* (CPCs) that were sampled to completion with the aim of contributing reliable data to the biogeography debate concerning microbes. To establish co-occurrence patterns, each CPC is connected to the most similar CPC according to the local ranking approach (see Fig 6). It can be seen that CPCs within the same oceanic region are more connected to each other than to CPCs of different regions. For example, in 10 out of 13 cases, the CPCs that are most similar to CPCs from the North- and South-Indian Ocean (NIO and SIO, respectively) originate from the same oceanic regions. In the case of the Red Sea (RS), all CPCs are connected to each other and of the seven CPCs in the Mediterranean Sea (MS), six are linked. Thus, both these marginal seas grow their unique characteristic CPCs despite their physical connection through the Suez Canal. In the Mediterranean Sea, only the Strait of Gibraltar CPC (MSG) is more similar to a South Atlantic CPC (SAO6) than to the other Mediterranean CPCs. This is not unexpected, since surface waters in the Strait of Gibraltar are characteristic Atlantic water masses [48]. Our data nicely demonstrate the transition of Atlantic CPCs into Mediterranean Sea CPCs.

Over the whole data set, 26 out of 35 links are connecting CPCs from exactly the same region. Combining NIO and SIO into a single region, the ratio improves to 29: 35. Thus, even though all oceanic regions are connected with each other through the meridional overturning circulation [49], which provides the opportunity for a circumglobal passive dispersal of microbial plankton, local diversity patterns are significantly more frequent than distant diversity patterns. This indicates a strong local species sorting, which contributes to the generation of a highly complex diversity. A global dispersal of microbes seems unlikely, even in the oceanic realm, where no physical barriers act as dispersal barriers (as would be the case for high mountain ranges on a continental scale). Thus, our analysis strongly supports the hypothesis that microorganisms (or at least CPCs) follow similar dispersal patterns and rules as macroorganisms [45].

## Conclusions and Discussion

In this article we considered the link assessment problem, the task of disentangling complex networks by distinguishing low-intensity real interactions from other spurious connections that may be erroneously included due to false observations and missing connections that may be incorrectly omitted. This problem is prevalent in most complex network settings based on real-world observations and has two predominant versions. In the global version, the overall most likely links are identified (i.e. links with the highest probability of being true). In the local version of the problem, links are assessed on a node-by-node basis to find the most probable neighbors for a given node. As such, link assessment has some important similarities and dissimilarities with other lines of current research. On the one hand, the local version of the problem is formally equivalent to the one-mode projection of bipartite graphs and can be directly applied in personalized recommendation [14, 15]. On the other hand, it extends link prediction, i.e. the problem of assessing the likelihood that two yet unconnected nodes will be connected in the future, given a snapshot of some dynamically evolving network. In link assessment, additionally to inferring missing links, edges that are present in the observed network are validated based on the likelihood that the two nodes should be connected given the surrounding network topology.

To find or develop a method that is applicable broadly and independently of the data set in question, we considered several data sets of interactions including a protein network from DIP, a social network from LiveJournal, and movie/TV networks from MovieLens and Netflix. On these data sets we compared a number of previously introduced similarity measures and then proposed a novel method, which is based on the statistical assessment of the number of common neighbors against an appropriate random graph model, the FDSM. We found that this degree-preserving random graph model is superior to other null models like the configuration or the hypergeometric model. Ranking node pairs by the empirical *p*-value in the FDSM and then braking ties by the *z*-score, the resulting z*-ranking is thus a simple, yet efficient way to benefit from the information contained in both test statistics. We showed that z* has the best consistent performance on these data sets. As a potential direction for future research, obtaining access to an even broader selection of benchmarks might allow disentangling the relationship between network structure and the performance of the individual measures. Eventually, such studies could be aimed at identifying network typologies that are constructed around the computationally least demanding measures that approach the top performance of z* most reliably and effectively.

Recently, attempts have been made to tackle the link prediction problem by combining multiple similarity measures through so-called ensemble methods (see for instance [50]). Accordingly, a common strategy is to use all available similarity measures as features in some prediction algorithm, such as logistic regression or support vector machines. However, these methods require the knowledge of a ground truth data set, the computation of several candidate measures, and many non-trivial modelling decisions throughout the process. This renders them useless in exploratory settings, where little data requirement and easy computation are invaluable. Thus, in this article, we focused on unsupervised methods and assessed their quality based on data set from very different domains.

Finally, we applied the method to a high-throughput sequence data set obtained from a global collection of microbial ocean plankton (data without ground truth) and explored a debated open question about the distribution of microbes on different scales. Based on this data, we found evidence that the global dispersal hypothesis for microbes should be rejected. Instead, the new method provides clear evidence that microbes follow similar biogeographic rules as higher animals and plants. This exploratory analysis, alongside the results of our benchmarks, shows that it is possible to refine data from noisy large-scale data sets by using a method which is only based on structural information. This indicates that the notion of homophily, which states that nodes with high similarity are more likely to form links, is much more universal than previously thought and even extends to non-human entities such as proteins and movies.

## Supporting Information

S1 Text Data preprocessing and ground truths. (PDF)

S2 Text Optimality in the link assessment problem. (PDF)

S3 Text Existing similarity measures and z*. (PDF)

S4 Text Ecological data. (PDF)

S1 Data Zip of all ground truth and available data sets. (ZIP)
