# Supplementary material for: Assessing Low-Intensity Relationships in Complex Networks
Source: PLoS One. 2016 Apr 20;11(4):e0152536. doi: 10.1371/journal.pone.0152536 (PMC4838277; doi:10.1371/journal.pone.0152536)
Supplement: S1 Text — (PDF) [file pone.0152536.s001.pdf]

S1 Text: Data preprocessing and ground truths

Protein–protein interaction network The protein–protein interaction (PPI) network that we use to compare the performance of the similarity measures in the link assessment problems is constructed for the unicellular model organism *Saccharomyces cerevisiae* yeast [1]. An empirical estimate of the interactome of this yeast contains $18,000 \pm 4,500$ interactions [2]. Other estimates assume over $30,000$ interactions between roughly $6,000$ proteins [3]. These large discrepancies in the estimations of the size of the interactome occur mainly due to two reasons:

1. The large proportion of false positives in the recorded interactions [4]. Several factors, such as the detection of biologically non-relevant interactions between proteins that never simultaneously co-occur *in vivo* [5], may favor false positives.
2. The incompleteness of available data on protein–protein interactions, as it is infeasible to test all possible interactions [6].

A network analytic approach holds promise for evaluating the reliability of interactions based on the assumption that the overall structure of the protein–protein interaction network is characteristic. Thus, the network structure provides evidence based on which we can detect false positive interactions and suggest candidate interactions for experimental testing. For a similar endeavor that is restricted to just a few node similarity measures, see Reference [7], while for an approach based on stochastic block models, see Reference [8].

To evaluate individual similarity measures, results of well-documented small-scale experiments can be used as reference [2]. Here we consider the full set of protein–protein interactions available for *Saccharomyces cerevisiae* on the public repository Database of Interacting Proteins (DIP) [9], release of August 18, 2012 (registration necessary; the data can be found under 'files', 'species-specific sets', under the tab called *S. cerevisiae.* The link's name is "Scere20120818"). The data integrates information from large- and small-scale experiments reported in the literature. After removing 318 self-interactions, the network contains $22,148$ interactions between $5,078$ proteins. The $3,543$ interactions that are classified as *dip-quality control core* and share at least one common neighbor constitute the `PPI` ground truth. We uploaded the file together with our other available data sets as supplemental information.

LiveJournal online social network On the blogging platform LiveJournal, individuals from a social network can explicitly state their group memberships. Groups are formed based on a common interest around which the group is organized. Yang and Leskovec provided a LiveJournal friendship network along with user-defined groups that contain at least three individuals [10]. As this network is prohibitively large ($34,681,189$ connections between $3,997,962$ individuals), we compare the similarity measures based on a sample. To generate this sample, we first select all groups of size ≤ 50. Here, our assumption is that larger groups do not allow a close relationship between the individuals. We then proceed by picking a starting group uniformly at random and continually increase the network through the addition of adjacent groups. Two groups are considered adjacent if they share at least one individual. The process is stopped once $1,000$ groups are selected and results in a network of $11,755$ individuals with a total of $80,023$ links.

To construct the `LiveJournal` ground truth, we consider that $126,515$ pairs of these individuals can be assumed to be similar based on shared group membership. To establish this similarity, we take into account membership in groups of size ≤ 50 in the *entire* data set. The sampled data as input data and the corresponding ground truth data was uploaded as supplemental information.

MovieLens data We also analyse a randomly selected sample extracted from the largest MovieLens data set (MovieLens 10M) [11]. The data were collected from the online recommender service MovieLens and made available by GroupLens Research. It contains films rated by users on a scale of 1 (dislike) to 5 (like). We sample by choosing a number of users uniformly at random and including in the sample all their ratings. We restrict this sample to positive ratings (i.e. having values of 4 and above) and then exclude all users who have less than 2 remaining ratings. The procedure results in $1,077,270$ ratings given to $9,153$ films by $15,185$ users and can be modeled by a bipartite graph.

We constructed a ground truth for this data based on the concept of film series. For this purpose, we match a comprehensive list of feature film series obtained from Wikipedia [12] to the films in the MovieLens data set. We consider two films to be similar if they belong to the same film series. This results in the ground truth called `MovieLens Movies` and contains 450 pairs of films. The ground truth was uploaded as supplemental information.

Netflix data Analogously to the MovieLens data, we extract a sample from the Netflix data set [13] which also contains ratings of films by users. The selection procedure is identical to the one described above; the resulting network contains $2,399,429$ ratings between $16,306$ films and $20,078$ users.

We create a ground truth of 904 feature film pairs in the same way as we did for the MovieLens data set (`Netflix Movies`). Additionally, as this data set contains ratings for TV shows, we build a second ground truth that relies on TV shows and consists of coherent groups of TV shows like *Friends* and *Star Trek* without their spin-offs (`Netflix TV Series`) [14, 15]. We do this by extracting all films with the keyword *season* in their title and connecting two seasons if they belong to the same TV show. The `Netflix TV Series` ground truth data set contains 951 pairs of TV shows.

The two ground truth data sets are contained in the uploaded supplemental information. The Netflix data itself was obtained from the Netflix prize (`http://netflixprize.com/`) website when it was still live. The regulations the authors signed back then do not allow to share the original data.

References

1. Uetz P, Giot L, Cagney G, Mansfield TA, Judson RS, Knight JR, et al. A comprehensive analysis of protein–protein interactions in *Saccharomyces cerevisiae*. Nature. 2000;403(6770):623–627.

2. Yu H, et al. High-quality binary protein interaction map of yeast interactome network. Science. 2008;322(5898):104–110.

3. Blow N. Untangling the protein web. Nature. 2009;460(7253):415–418.

4. Prieto C, Rivas JDL. Structural domain–domain interactions: Assessment and comparison with protein–protein interaction data to improve the interactome. Proteins. 2009;78(1):109–117.

5. Giot L, et al. A protein interaction map of *Drosophila melanogaster*. Science. 2003;302(5651):1727–1736.

6. Shoemaker BA, Panchenko AR. Deciphering Protein–Protein Interactions. Part I. Experimental Techniques and Databases. PLOS Computational Biology. 2007;3(3):e42.

7. Goldberg DS, Roth FP. Assessing experimentally derived interactions in a small world. Proceedings of the National Acedemy of Sciences. 2003;100(8):4372–4376.

8. Guimerà R, Sales-Pardo M. Missing and spurious interactions and the reconstruction of complex networks. Proceedings of the National Academy of Sciences. 2009;106(52):22073–22078.

9. Database of Interacting Proteins (DIP);. Accessed March 11, 2014. http://dip.doe-mbi.ucla.edu/dip/Download.cgi.

10. Yang J, Leskovec J. Defining and evaluating network communities based on ground-truth. In: Proceedings of the ACM SIGKDD Workshop on Mining Data Semantics. 3; 2012. .

11. MovieLens GroupLens;. Accessed March 11, 2014. http://grouplens.org/datasets/movielens/.

12. Film series at Wikipedia;. Accessed March 11, 2014. http://en.wikipedia.org/wiki/Lists_of_film_series.

13. The Netflix Prize;. Accessed March 11, 2014. http://www.netflixprize.com/.

14. Zweig KA. How to Forget the Second Side of the Story: A New Method for the One-Mode Projection of Bipartite Graphs. In: Proceedings of the second International Conference on Advances in Social Network Analysis and Mining. IEEE Computer Society; 2010. p. 200–207.

15. Zweig KA, Kaufmann M. A systematic approach to the one-mode projection of bipartite graphs. Social Network Analysis and Mining. 2011;1(3):187–218.
