# Supplementary material for: Assessing Low-Intensity Relationships in Complex Networks
Source: PLoS One. 2016 Apr 20;11(4):e0152536. doi: 10.1371/journal.pone.0152536 (PMC4838277; doi:10.1371/journal.pone.0152536)

S2 Text: Optimality in the link assessment problem

Graph theoretic definitions

$G = (V, E)$ denotes an undirected graph with a set of nodes V and a set of links $E \subseteq V \times V$ that represent given relationships between the nodes. $\Gamma(v)$ denotes the *set of neighbors* of node v, i.e. all nodes w such that $(v, w) \in E$. The *degree* $d(v)$ of node v is defined as the number of its neighbors, i.e. $d(v) = |\Gamma(v)|$. A graph is said to be bipartite if the node set V can be partitioned into two sets L and R, such that each link connects a node from L with a node from R, i.e. $E \subseteq L \times R$. A graph's *degree sequence* is defined as the ordered sequence of degrees of its nodes.

Optimality in the link assessment problem

Proposition: *A globally optimal similarity measure is necessarily locally optimal as well, but not vice versa.*
Proof: Let s be globally optimal and let $v, w, w' \in V$. If $(v, w) \in E_{GT}$ and $(v, w') \notin E_{GT}$ then it follows by the definition of global optimality that $s(v, w) \geq s(v, w')$. Since the above is true for any choice of v, w, and w', it follows that s is locally optimal. To show that a locally optimal measure is not necessarily globally optimal, consider the following counterexample: The solid line denotes a ground truth link between v and u, the dashed lines denote links that are not in the ground truth. Observe that the similarity measure generating these weights is locally optimal, as it results in the correct identification of the ground truth link (v, u). In a global approach however, the link (w, x) would erroneously be selected. Therefore, the measure is not globally optimal.

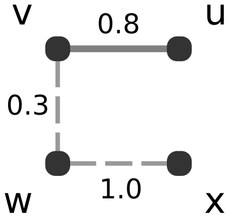

Supplement: S2 Text — (PDF) [file pone.0152536.s002.pdf]
