# Supplementary material for: Assessing Low-Intensity Relationships in Complex Networks
Source: PLoS One. 2016 Apr 20;11(4):e0152536. doi: 10.1371/journal.pone.0152536 (PMC4838277; doi:10.1371/journal.pone.0152536)
Supplement: S3 Text — (PDF) [file pone.0152536.s003.pdf]

S3 Text: Existing similarity measures and z^*

Structural node similarity

The notion of measuring the level of similarity between nodes is central to a variety of problems in network science. Formulated on a global level in terms of the question *How similar are two given nodes?*, similarity is central to clustering. Termed on a local level, i.e. *Which other nodes are most similar to a particular node?*, it represents a key issue in recommendation. Despite its broad applicability, node similarity is a concept that received little attention (for notable exceptions see References [1, 2]) when compared to several other network measures such as the clustering coefficient [3], degree distribution [4], or centrality indexes [5–8].

Two nodes can be alike in many respects such as shared external factors or a similar position in the network. In this paper, we concentrate on node similarity based solely on network structure, meaning that no additional node attributes are taken into account (such as the age, gender, location, or occupation of individuals in a social network or the 3D structure of proteins in a protein–protein interaction network). In the network science literature, the most common approach to constructing mathematical measures for the quantification of similarity is termed *structural equivalence* and determines the similarity between nodes in a network based on their position [2, 9, 10]. Specifically, two nodes are considered to be similar if they share the same neighbors. This general idea relies on two basic assumptions: 1. the structure of the network reflects real information about the nodes and the structural similarity is thus well-suited for any network where the function or role of a node is related to its structural surroundings, and 2. links in the network indicate fundamental similarity between the nodes they connect. Given these two assumptions, the structural equivalence-based notion of similarity is appropriate for instance in the identification of functional categories or in functional prediction.

Similarity measures introduced previously

In the following, we review a selection of the most well-known similarity measures, which are based on the idea of structural equivalence. We use these as predictors in the *link assessment problem*, i.e. they are scores that enable us to rank the pairs of nodes by their reliability given the structure of the network. We formulate each measure both for non-bipartite and bipartite graphs, which are undirected and unweighted and thus have a binary adjacency matrix that encodes the presence or absence of a link between two nodes. Table 1 summarizes the considered measures.

Number of common neighbors Based on the intuition about various types of networks, the most basic indicator of the similarity between two nodes is the number of their common neighbors. For instance, two individuals in a social network who share several acquaintances are likely to have similar domicile, age, or activities [11, p. 77–83]; two films that are liked by the same people might be similar in terms of story, cast, and style [12]; two genes regulated by the same transcription factors can be assumed to share sequence similarity or functional role [13]; and two scientific papers that are often cited together may deal with related topics [2, p. 70].

The number of common neighbors in a graph theoretical sense is closely related to the so-called *co-occurrence* defined for diverse relational data. Examples of

co-occurrence are for instance terms that appear in the same document or sentence in text mining [14, 15], species that inhabit the same location in ecology [16, ch. 7], or items that are purchased together in market basket analysis [17, p. 299–302].

The number of common neighbors of nodes v and w is the intersection of their neighborhoods and can be computed as the scalar product of their respective rows in the $(0, 1)$-valued adjacency matrix A:

$$n(v, w) := |\Gamma(v) \cap \Gamma(w)| = A_v \cdot A_w = \sum_{u=1}^{n} A_{vu} A_{wu} \tag{1}$$

where $\Gamma(v)$ is the neighbor set of node v and $|\cdot|$ denotes the cardinality of the set.

A key property of the number of common neighbors is that it is bounded by the smaller degree of the involved nodes:

$$0 \leq n(v, w) \leq \min\{d(v), d(w)\} \tag{2}$$

Thus, the number of common neighbors has the shortcoming that it may assume larger values for high degree nodes sharing only a small percentage of their neighbors than for small degree nodes with a relatively large neighborhood overlap. The majority of wide-spread similarity measures thus normalizes the number of common neighbors in some way to compensate for the degrees such that the similarity is 1 for perfectly overlapping neighborhoods. A prominent example of this is the Jaccard index.

Jaccard index One of the oldest similarity measures was proposed by Paul Jaccard to measure the likeness of two sets [18] and it has been heavily used ever since (see for example Reference [19]). In a network setting, the Jaccard index normalizes the size of the intersection of the neighbor sets of nodes v and w by the cardinality of their union:

$$Jaccard(v, w) := \frac{|\Gamma(v) \cap \Gamma(w)|}{|\Gamma(v) \cup \Gamma(w)|} \tag{3}$$

where $\Gamma(v)$ is the neighbor set of node v. Expressed in terms of the number of common neighbors and the degrees, the above formula can be rewritten as:

$$Jaccard(v, w) = \frac{n(v, w)}{d(v) + d(w) - n(v, w)} \tag{4}$$

The Jaccard index assumes values from the interval $[0, 1]$ and reaches its optimal value of 1 when the two nodes have exactly the same neighbors. Note that it is undefined for nodes with degree 0. In this case, it can be explicitly set to 0.

Cosine similarity The cosine similarity or Salton's cosine [20] gives the angular cosine distance between the n-dimensional vectors $\vec{x}, \vec{y} \in \mathbb{R}^n$:

$$cosine(\vec{x}, \vec{y}) := \frac{\vec{x} \cdot \vec{y}}{\|\vec{x}\|_2 \, \|\vec{y}\|_2} \tag{5}$$

where $\cdot$ denotes the scalar product of two vectors and $\|\cdot\|_2$ is the Euclidean norm. Note that the cosine similarity is invariant against scaling of the vectors:

$$cosine(\alpha\,\vec{x}, \vec{y}) = cosine(\vec{x}, \vec{y}) \;\; \forall\, \vec{x}, \vec{y} \in \mathbb{R}^n \text{ and } \alpha \in \mathbb{R}_+ \tag{6}$$

Therefore, it is extensively used in data mining, where the relative distribution of the feature values needs to be taken into account. Its applications range from document comparison to collaborative filtering (see for instance References [21, 22]).

The cosine similarity of nodes v and w is computed from their corresponding rows in the adjacency matrix:

$$cosine(v,w) = \frac{A_v \cdot A_w}{\|A_v\|_2 \, \|A_w\|_2} = \frac{n(v,w)}{\sqrt{d(v)d(w)}} \tag{7}$$

Accordingly, the cosine similarity equals the number of common neighbors $n(v,w)$ when normalized by the geometric average of the degrees $d(v)$ and $d(w)$. Its values lie in the range from 0 to 1, with 1 indicating a perfect overlap between the neighborhoods. As in the case of the Jaccard index, the cosine is also undefined for zero vectors and requires an explicit definition for this case.

Adamic-Adar similarity The *AdamicAdar* measure [23, 24] is defined as the weighted sum of the degrees of v and w's common neighbors u:

$$AdamicAdar(v,w) := \sum_{u \in \Gamma(v) \cap \Gamma(w)} \frac{1}{log(d(u))} \tag{8}$$

Due to the used logarithmic weighting scheme, common neighbors of v and w with small degrees contribute more to the similarity of v and w. The measure takes its values from $\mathbb{R}_+$ with larger values indicating a higher similarity.

Resource allocation index The *Resource allocation index* (rai) is similar to the Adamic-Adar index. It is motivated by the resource allocation dynamics on complex systems by assuming that each node has one unit of some ressource which is evenly distributed among all its neighbors. The approach thus defines the similarity of two nodes by the sum of the inverse degrees of their common neighbors [25]:

$$rai(v,w) := \sum_{u \in \Gamma(v) \cap \Gamma(w)} \frac{1}{d(u)} \tag{9}$$

Leverage under the simple independence model An intuitive way of correcting the observed number of common neighbors is to account for the expected number of common neighbors the two nodes *would* have if both would choose their neighbors purely at random. This approach is incorporated in the measure called *leverage*, which subtracts from the observed number of common neighbors the expected value under this *simple independence model* (SIM).

The same measure is used in market basket analysis to investigate patterns of the form: *If customers bought a set of products* $\mathcal{Q}_1$ *then they also purchased the set of products* $\mathcal{Q}_2$. For two disjoint sets of products $\mathcal{Q}_1$ and $\mathcal{Q}_2$, this pattern is formulated as a so-called *association rule* $\mathcal{Q}_1 \rightarrow \mathcal{Q}_2$, meaning that if a data set contains the products in $\mathcal{Q}_1$ then it also contains the products in $\mathcal{Q}_2$. The leverage then serves to assess the meaningfulness of such implications [26] and is defined as the difference between the joint probability of $\mathcal{Q}_1$ and $\mathcal{Q}_2$'s occurrence $P(\mathcal{Q}_1, \mathcal{Q}_2)$ (i.e. their co-occurrence) and their expected probability if they had appeared independently from each other, i.e. under the simple independence model:

$$P(\mathcal{Q}_1 \rightarrow \mathcal{Q}_2) := P(\mathcal{Q}_1, \mathcal{Q}_2) - P(\mathcal{Q}_1)P(\mathcal{Q}_2) \tag{10}$$

In the context of graphs, the leverage of nodes v and w is computed analogously:

$$lev[SIM](v,w) = \frac{1}{|V|}\left(n(v,w) - \frac{d(v)d(w)}{|V|}\right), \tag{11}$$

where $|V|$ denotes the number of nodes in the graph and the subtracted term $d(v)d(w)/|V|$ represents the expected number of common neighbors under the simple independence model [2, p. 214]. Note that in the case of bipartite graphs, the expected number of common neighbors of nodes $v, w \in L$ is $d(v)d(w)/|R|$, where $|R|$ denotes the number of nodes in the opposite set. Thus, their leverage is:

$$lev[SIM](v,w) = \frac{1}{|R|}\left(n(v,w) - \frac{d(v)d(w)}{|R|}\right) \tag{12}$$

As Zweig et al. showed, the independence assumption implied by the SIM is overly simplistic for real-world applications [27,28]. From a formal point of view, $lev[SIM]$ is equivalent to the *covariance* [2, p. 215]. The Pearson correlation coefficient as presented next offers a further normalization of this measure.

Leicht-Holme-Newman index The *Leicht-Holme-Newman index* (lhn) is based on the same simple null model and is defind as the ratio between the observed and expected number of common neighbors [1]:

$$lhn(v,w) := \frac{n(v,w)}{d(v)d(w)} \tag{13}$$

The Leicht-Holme-Newman index assigns high similarity values to those pairs of nodes which have many common neighbors compared to the expected number of neighbors.

Pearson correlation coefficient The Pearson correlation or Pearson product-moment correlation is widely used to measure linear dependency between vectors and allows for several interpretations [29]. It is commonly used in traditional collaborative filtering approaches [30, p. 17] and recently also in systems biological analysis (see for example Reference [31]).

The Pearson correlation is often employed to measure node similarity based on the corresponding rows of the adjacency matrix and is simply computed by rescaling the covariance [2, p. 214–215]:

$$Pearson(v,w) := \frac{cov(v,w)}{\sigma[v]\sigma[w]}, \tag{14}$$

where $\sigma[v]$ denotes the standard deviation of the adjacency row of node v. Substituting the covariance with the equivalent leverage as computed in Equation 11, we obtain the Pearson correlation coefficient:

$$Pearson(v,w) = \frac{1}{|V|\ \sigma[v]\sigma[w]}\left(n(v,w) - \frac{d(v)d(w)}{|V|}\right) \tag{15}$$

The values of the Pearson correlation lie in the interval $[-1, 1]$ with $r \approx 1$ being a strong positive correlation and $r \approx -1$ meaning a strong negative correlation. The Pearson correlation for bipartite graphs is:

$$Pearson(v,w) = \frac{1}{|R|\ \sigma[v]\sigma[w]}\left(n(v,w) - \frac{d(v)d(w)}{|R|}\right) \tag{16}$$

Note that for centered adjacency rows, i.e. where $\langle A_v \rangle = \langle A_w \rangle = 0$, the Pearson correlation and the cosine similarity are equivalent [29]. Here, $\langle \cdot \rangle$ denotes the average.

Hypergeometric coefficient The cumulative hypergeometric distribution has been used extensively to measure the significance of the number of common neighbors in biochemistry and systems biology (see for instance References [32–35]). The hypergeometric distribution assumes a null model in which the neighborhoods of the two nodes are independent and the common neighbors are chosen without replacement. It is computed from the degrees of the two nodes and the total number of nodes as follows:

$$p[hypergeom](v,w) := \sum_{c=n(v,w)}^{\min\{d(v),d(w)\}} \frac{\binom{d(v)}{c}\binom{|V|-d(v)}{d(w)-c}}{\binom{|V|}{d(w)}} \tag{17}$$

The sum gives the probability of obtaining a number of common neighbors which is at least as large as the observed number of common neighbors and can thus be interpreted as a p-value.

Note that in the case of bipartite graphs, the hypergeometric coefficient for two nodes $v, w \in L$ is:

$$p[hypergeom](v,w) = \sum_{c=n(v,w)}^{\min\{d(v),d(w)\}} \frac{\binom{d(v)}{c}\binom{|R|-d(v)}{d(w)-c}}{\binom{|R|}{d(w)}} \tag{18}$$

Empirical p-value under the fixed degree sequence model The underlying null models of the previous measures contain independence assumptions that are potentially overly simplistic in the case of real-world networks [28]. A more realistic model is the *fixed degree sequence model* (FDSM), which contains all graphs with a given degree sequence [36–42]. A popular method of assessing the statistical significance of the observed number of common neighbors of v and w based on this model is to count the fraction of graphs in which this value is at least as large as the observed value $n(v,w)$:

$$p[FDSM](v,w) := \frac{|\{G \in \mathcal{H} \mid n_G(v,w) \geq n(v,w)\}|}{|\mathcal{H}|}, \tag{19}$$

where $n_G(v,w)$ is the number of common neighbors of v and w in a graph $G \in \mathcal{G}$ from the ensemble $\mathcal{G}$ that contains all graphs with the same degree sequence as the observed graph (see for instance Reference [43]). Since the graph ensemble cannot be exhaustively enumerated, an empirical p-value is computed based on the random sample $\mathcal{H} \subset \mathcal{G}$. This empirical measure approximates the true p-value. The observed number of common neighbors is thus compared to the expected number of common neighbors in $\mathcal{H}$.

The smaller the p-value, the more unlikely it is that the two nodes have the observed number of common neighbors in a random graph and thus the more significant the observation.

The numerical estimation of the p-values in the tail of the distribution (i.e. where many of our points of interest lie) requires a large sample [44]. In contrast, calculating the z-scores as described next, requires sampling of only the first two moments of the distribution, namely the average and the standard deviation.

z-score under the fixed degree sequence model Assuming Poisson distributed numbers of common neighbors in the graphs of the FDSM model, a Gaussian distribution is a good fit for nodes with large degree and thus the z-score can be used as test statistic instead of the empirical p-value. The z-score quantifies the deviation from the sample average in units of standard deviation. Applied to the number of common neighbors, we obtain:

$$z[FDSM](v,w) := \frac{n(v,w) - \langle n_G(v,w)\rangle}{\sigma[n_G(v,w)]}, \tag{20}$$

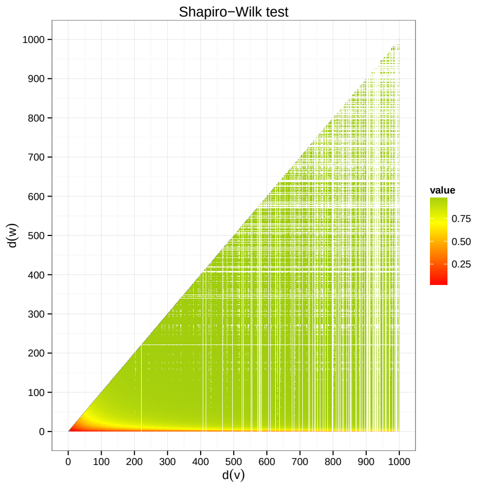

Figure 1. Shapiro-Wilk normality test for the number of common neighbors of pairs of nodes v and w in a sample of $1,000$ graphs taken from the fixed degree sequence model. Shown are test results for pairs with degrees $d(v), d(w) \leq 1,000$. The selected pairs of nodes have at least one common neighbor in the `Netflix` data set. A value close to 1 is a good fit for the sampled distribution of the number of common neighbors.

where the notation is consistent with that in Equation 19, $\langle \cdot \rangle$ denotes the sample average, and $\sigma[\cdot]$ denotes the sample standard deviation.

Our tests on the `Netflix` data set indicate that the distribution of the number of common neighbors can be approximated by a normal distribution for large degree nodes. Fig. 1 shows the results of a Shapiro-Wilk normality test and in Fig. 2 we present a normal fit for selected degree pairs.

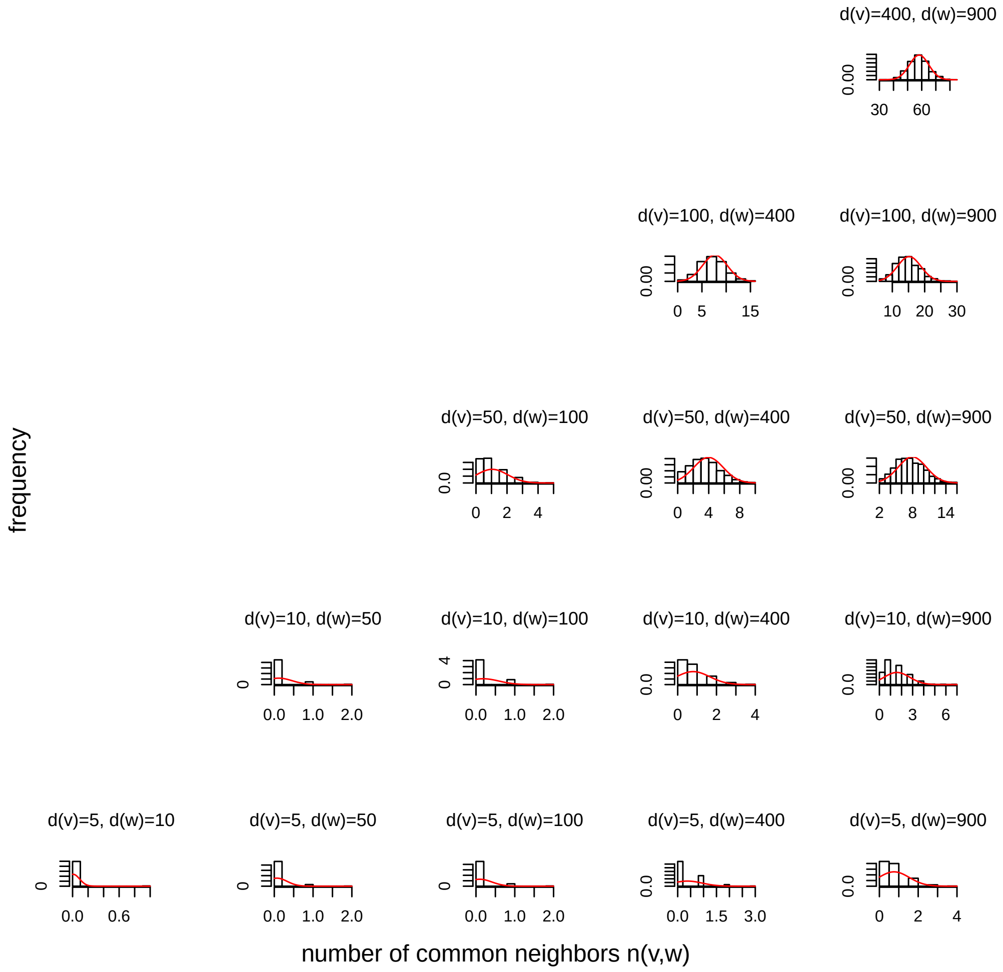

Figure 2. Histogram of the number of common neighbors $n(v, w)$ for selected pairs v and w with the specified degrees. The values shown in the histograms were taken from $1,000$ samples of the fixed degree sequence model. The red curve represents a fitted normal distribution.

The newly introduced presorted z-score, z^*

For obtaining accurate p-values, a large sample is needed. Thus, a p-value that is near 0 can indicate genuine significance or the fact that the number of samples is not sufficient. In the range of low p-values, it can thus be rewarding to use the z-score, because it differentiates better than the p-value when computed based on a sample of the same size. Therefore, we propose to assess the similarity of nodes based on the combination of these two measures. Let $<_p$ and $<_z$ denote the order of the node pairs according to p-value and z-score, respectively. Then, for the node pairs (v, w) and (x, y) we define the order $<_{z^*}$ as:

$$(v, w) <_{z^*} (x, y) \iff \{\, (v, w) <_p (x, y) \,\} \vee \{\, (v, w) =_p (x, y) \wedge (v, w) <_z (x, y) \,\} \qquad (21)$$

This ranking, called z^*, therefore orders the pairs of nodes first by p-value and breaks the ties according to an ordering by z-score to obtain the final ranking The idea is that,

similarity measure	formula	range	optimal value
$Jaccard$	$\frac{n(v,w)}{d(v)+d(w)-n(v,w)}$	$[0,1]$	1
$cosine$	$\frac{n(v,w)}{\sqrt{d(v)d(w)}}$	$[0,1]$	1
$AdamicAdar$	$\sum_{u\in\Gamma(v)\cap\Gamma(w)} \frac{1}{log(d(u))}$	$\mathbb{R}_+$	∞
rai	$\sum_{u\in\Gamma(v)\cap\Gamma(w)} \frac{1}{d(u)}$		
$\boldsymbol{lev[SIM]}$	$\frac{1}{\lvert V\rvert}\left(n(v,w)-\frac{d(v)d(w)}{\lvert V\rvert}\right)$	$[-1,1]$	1
lhn	$\frac{n(v,w)}{d(v)d(w)}$		
$\boldsymbol{Pearson}$	$\frac{cov(v,w)}{\sigma[v]\sigma[w]}$	$[-1,1]$	1
$\boldsymbol{p[hypergeom]}$	$\sum_{c=n(v,w)}^{\min\{d(v),d(w)\}} \frac{\binom{d(v)}{c}\binom{\lvert V\rvert-d(v)}{d(w)-c}}{\binom{\lvert V\rvert}{d(w)}}$	$[0,1]$	0
$p[FDSM]$	$\frac{\lvert\{G\in\mathcal{H} \mid cooc_G(v,w)\geq cooc(v,w)\}\rvert}{\lvert\mathcal{H}\rvert}$	$[0,1]$	0
$z[FDSM]$	$\frac{cooc(v,w)-\langle cooc_G(v,w)\rangle}{\Gamma[cooc_G(v,w)]}$	$\mathbb{R}$	∞
z^*	see text	–	–

Table 1. Summary of the presented similarity measures, all of which are based on the number of common neighbors of two given nodes. Bold measures have a slightly different formula when adapted to bipartite graphs (see text for details).

in the computation of the empirical p-value based on a sample of τ graphs, any combination of common neighbors that occur with a probability of less than $1/\tau$ is likely to be observed either just once in the sample or not at all. In the first case, its real probability is overestimated, in the second it is underestimated. A more realistic measure in this case is the empirical z-score, since this measure approximates only the first and second moments of the distribution of the number of common neighbors throughout the samples. In other words, a p-value that is near 0 can indicate genuine significance or the fact that the number of samples is not sufficient. In the range of low p-values, it can thus be rewarding to use the z-score, because it differentiates better than the p-value when computed based on a sample of the same size. Note that z^* does not provide a similarity score for two nodes, but results in a ranking of the pairs of nodes.

References

1. Leicht EA, Holme P, Newman MEJ. Vertex similarity in networks. Physical Review E. 2006;73(2):026120.
2. Newman MEJ. Networks. An introduction. Oxford; 2010.
3. Watts DJ, Strogatz SH. Collective dynamics of 'small-world' networks. Nature. 1998;393(6684):440–442.
4. Barabási AL, Albert R. Emergence of Scaling in Random Networks. Science. 1999;286(5439):509–512.

5. Koschützki D, Lehmann K, Peeters L, Richter S, Tenfelde-Podehl D, Zlotowski O. Centrality Indices. In: Brandes U, Erlebach T, editors. Network Analysis: Methodological foundations. Springer; 2005. p. 16–61.

6. Jacob R, Koschützki D, Lehmann K, Peeters L, Tenfelde-Podehl D. Algorithms for Centrality Indices. In: Brandes U, Erlebach T, editors. Network Analysis: Methodological foundations. Springer; 2005. p. 62–82.

7. Koschützki D, Lehmann K, Tenfelde-Podehl D, Zlotowski O. Advanced Centrality Concepts. In: Brandes U, Erlebach T, editors. Network Analysis: Methodological foundations. Springer; 2005. p. 83–110.

8. Borgatti SP, Everett MG. A graph-theoretic perspective on centrality. Social Networks. 2006;28(4):466–484.

9. Lorrain F, White HC. Structural equivalence of individuals in social networks. The Journal of Mathematical Sociology. 1971;1(1):49–80.

10. Borgatti SP. 2-mode concepts in social network analysis. In: Meyers RA, editor. Encyclopedia of Complexity and System Science. New York: Springer; 2009. p. 8279–8291.

11. Easley D, Kleinberg J. Networks, Crowds, and Markets: Reasoning about a Highly Connected World. New York: Cambridge University Press; 2010.

12. Adomavicius G, Tuzhilin A. Toward the next generation of recommender systems: a survey of the state-of-the-art and possible extensions. Knowledge and Data Engineering, IEEE Transactions on. 2005;17(6):734–749.

13. Yu H, Luscombe NM, Qian J, Gerstein M. Genomic analysis of gene expression relationships in transcriptional regulatory networks. TRENDS in Genetics. 2003;19(8):422–427.

14. Cohen AM, Hersh WR, Dubay C, Spackman K. Using co-occurrence network structure to extract synonymous gene and protein names from MEDLINE abstracts. BMC Bioinformatics. 2005;6(1):103.

15. Feldman R, Sanger J. The Text Mining Handbook: Advanced Approaches in Analyzing Unstructured Data. New York: Cambridge University Press; 2007.

16. Gotelli NJ, Graves GR. Null Models in Ecology. Washington D.C.: Smithsonian Institution Press; 1996.

17. Linoff GS, Berry MJ. Data Mining Techniques: For Marketing, Sales, and Customer Relationship Management. 2nd ed. Wiley Publishing, Inc.; 2004.

18. Jaccard P. Distribution de la flore alpine dans le bassin des Dranses et dans quelques régions voisines. Bulletin de la Société Vaudoise des Sciences Naturelles. 1901;37:241–272.

19. Wolf YI, Karev G, Koonin EV. Scale-free networks in biology: new insights into the fundamentals of evolution? Bioessays. 2002;24(2):105–109.

20. Salton G, McGill MJ. Introduction to Modern Information Retrieval. New York, NY, USA: McGraw-Hill, Inc.; 1986.

21. Mane KK, Börner K. Mapping topics and topic bursts in PNAS. Proceedings of the National Acedemy of Sciences. 2004;101(suppl 1):5287–5290.

22. Linden G, Smith B, York J. Amazon.com Recommendations. Item-to-Item Collaborative Filtering. IEEE Internet Computing. 2003;7(1):76–80.

23. Adamic LA, Adar E. Friends and neighbors on the web. Social Networks. 2003;25(3):211–230.

24. Liben-Nowell D, Kleinberg J. The link-prediction problem for social networks. Journal of the American Society for Information Science and Technology. 2007;58(7):1019–1031.

25. Zhou T, Lü L, Zhang YC. Predicting missing links via local information. The European Physical Journal B. 2009;71(4):623–630.

26. Tan PN, Kumar V, Sivastava J. Selecting the right objective measure for association analysis. Information Systems. 2004;29(4):293–313.

27. Zweig KA. How to Forget the Second Side of the Story: A New Method for the One-Mode Projection of Bipartite Graphs. In: Proceedings of the second International Conference on Advances in Social Network Analysis and Mining. IEEE Computer Society; 2010. p. 200–207.

28. Zweig KA, Kaufmann M. A systematic approach to the one-mode projection of bipartite graphs. Social Network Analysis and Mining. 2011;1(3):187–218.

29. Rodgers JL, Nicewander WA. Thirteen ways to look at the correlation coefficient. The American Statistician. 1988;42(1):59–66.

30. Xu G, Zhang Y, Li L. Web mining and social networking. Techniques and applications. New York: Springer; 2011.

31. Marco A, Konikoff C, Karr TL, Kumar S. Relationship between gene co-expression and sharing of transcription factor binding sites in *Drosophila melanogaster*. Bioinformatics. 2009;25(19):2473–2477.

32. Travazoie S, Hughes JD, Campbell MJ, Cho RJ, Church GM. Systematic determination of genetic network architecture. Nature Genetics. 1999;22(3):281–285.

33. Sudarsanam P, Pilpel Y, Church GM. Genome-wide co-occurrence of promoter elements reveals a *cis*-regulatory cassette of rRNA transcription motifs in *Saccharomyces cerevisiae*. Genome Research. 2002;12(11):1723–1731.

34. Goldberg DS, Roth FP. Assessing experimentally derived interactions in a small world. Proceedings of the National Acedemy of Sciences. 2003;100(8):4372–4376.

35. Sun J, Gong X, Purow B, Zho Z. Uncovering microRNA and transcription factor mediated regulatory networks in glioblastoma. PLOS Computational Biology. 2012;8(7):e1002488.

36. Brualdi RA. Matrices of zeros and ones with fixed row and column sum vectors. Linear Algebra and its Applications. 1980;33:159–231.

37. Rao AR, Jana R, Bandyopadhyay S. A Markov chain Monte Carlo method for generating random (0,1)-matrices with given marginals. The Indian Journal of Statistics. 1996;58(Series A):225–242.

38. Gotelli NJ. Null model analysis of species co-occurrence patterns. Ecology. 2000;81(9):2606–2621.

39. Newman MEJ, Strogatz SH, Watts DJ. Random graphs with arbitrary degree distributions and their applications. Physical Review E. 2001;64(2):026118.

40. Maslov S, Sneppen K. Specificity and stability in topology of protein networks. Science. 2002;296(5569):910–913.

41. Cobb GW, Chen YP. An application of Markov Chain Monte Carlo to community ecology. The American Mathematical Monthly. 2003;110(4):265–288.

42. Butts CT. Social network analysis: A methodological introduction. Asian Journal of Social Psychology. 2008;11(1):13–41.

43. Yeger-Lotem E, Sattath S, Kashtan N, Itzkovitz S, Milo R, Pinter RY, et al. Network motifs in integrated cellular networks of transcription–regulation and protein–protein interaction. Proceedings of the National Academy of Sciences. 2004;101(16):5934–5939.

44. Traud AL, Kelsic ED, Mucha PJ, Porter MA. Comparing Community Structure to Characteristics in Online Collegiate Social Networks. SIAM Review. 2011;53(3):526–543.
