# Supplementary material for: Assessing Low-Intensity Relationships in Complex Networks
Source: PLoS One. 2016 Apr 20;11(4):e0152536. doi: 10.1371/journal.pone.0152536 (PMC4838277; doi:10.1371/journal.pone.0152536)
Supplement: S4 Text — (PDF) [file pone.0152536.s004.pdf]

# S4 Text: Ecological data

The analysed data contains roughly 4.5 million taxonomic marker gene sequences (hypervariable V9 region of the small subunit ribosomal DNA, SSU rDNA) from planktonic ciliates, a group of unicellular eukaryotes, essential for ecosystem functioning [1]. Samples were collected during the TARA Oceans project [2] in different oceanic regions, including the Mediterranean Sea (MS), the Red Sea (RS), the North and South Indian Ocean (NIO and SIO), the South Pacific Ocean (SPO), the South Atlantic Ocean (SAO), and the Southern Ocean (SO). Here we consider only the 35 surface water samples. Sequences were obtained as described in De Vargas et al. [3], then processed and binned into operational taxonomic units (OTUs, based on 97% sequence similarity) as described in Stoeck et al. [4]. Diversity partitioning of the obtained OTUs in the sampled oceanic regions was then analysed with the local link assessment procedure based on $z^*$.

## References

1. Barber R. OCEANS: Picoplankton do some heavy lifting. Science. 2007;315:777–778.

2. Karsenti E, Acinas S, Bork P, Bowler C, Vargas CD, Raes J, et al. A Holistic Approach to Marine Eco-Systems Biology. PLOS Biology. 2011;9(10):e1001177.

3. de Vargas C, Audic S, Henry N, Decelle J, Mahé F, Logares R, et al. Eukaryotic plankton diversity in the sunlit ocean. Science. 2015;348(6237).

4. Stoeck T, Bass D, Nebel M, Christen R, Jones MD, Breiner HW, et al. Multiple marker parallel tag environmental DNA sequencing reveals a highly complex eukaryotic community in marine anoxic water. Molecular Ecology. 2010;19:21–31.
